# Early-phase changes of extravascular lung water index as a prognostic indicator in acute respiratory distress syndrome patients

**DOI:** 10.1186/s13613-014-0027-7

**Published:** 2014-08-13

**Authors:** Takashi Tagami, Toshiaki Nakamura, Shigeki Kushimoto, Ryoichi Tosa, Akihiro Watanabe, Tadashi Kaneko, Hidetada Fukushima, Hiroshi Rinka, Daisuke Kudo, Hideaki Uzu, Akira Murai, Makoto Takatori, Hiroo Izumino, Yoichi Kase, Ryutarou Seo, Hiroyuki Takahashi, Yasuhide Kitazawa, Junko Yamaguchi, Manabu Sugita, Hiroyuki Takahashi, Yuichi Kuroki, Takashi Kanemura, Kenichiro Morisawa, Nobuyuki Saito, Takayuki Irahara, Hiroyuki Yokota

**Affiliations:** 1 Department of Emergency and Critical Care Medicine, Nippon Medical School, 1-1-5 Sendagi, Bunkyo-ku, Tokyo 113-8603, Japan; 2 Department of Clinical Epidemiology and Health Economics, School of Public Health, Graduate School of Medicine, The University of Tokyo, Tokyo 113-8654, Japan; 3 Intensive Care Unit, Nagasaki University Hospital, Nagasaki 852-8501, Japan; 4 Division of Emergency Medicine, Tohoku University Graduate School of Medicine, Miyagi 986-2242, Japan; 5 Department of Emergency and Critical Care Medicine, Aizu Chuo Hospital, Fukushima 965-8611, Japan; 6 Advanced Medical Emergency and Critical Care Center, Yamaguchi University Hospital, Yamaguchi 755-8505, Japan; 7 Department of Emergency and Critical Care Medicine, Nara Medical University, Nara 634-8522, Japan; 8 Emergency and Critical Care Medical Center, Osaka City General Hospital, Osaka 534-0021, Japan; 9 Department of Emergency and Critical Care Medicine, Kurume University School of Medicine, Fukuoka 830-0011, Japan; 10 Department of Emergency and Critical Care Medicine, Fukuoka University Hospital, Fukuoka 814-0180, Japan; 11 Department of Anesthesia and Intensive Care, Hiroshima City Hospital, Hiroshima 730-8518, Japan; 12 Advanced Emergency and Critical Care Center, Kansai Medical University Takii Hospital, Osaka 570-8507, Japan; 13 Department of Critical Care Medicine, Jikei University School of Medicine, Tokyo 105-8461, Japan; 14 Intensive Care Unit, Kobe City Medical Center General Hospital, Hyogo 650-0046, Japan; 15 Shock Trauma and Emergency Medical Center, Tokyo Medical and Dental University Hospital of Medicine, Tokyo 113-8519, Japan; 16 Department of Emergency and Critical Care Medicine, Kansai Medical University, Osaka 570-8506, Japan; 17 Division of Emergency and Critical Care Medicine, Department of Acute Medicine, Nihon University School of Medicine, Tokyo 173-8610, Japan; 18 Department of Emergency and Critical Care Medicine, Juntendo University Nerima Hospital, Tokyo 177-8521, Japan; 19 Department of Intensive Care Medicine, Saiseikai Yokohamashi Tobu Hospital, Kanagawa 230-8765, Japan; 20 Department of Emergency and Critical Care Medicine, Social Insurance Chukyo Hospital, Aichi 457-8510, Japan; 21 Emergency and Critical Care Medicine, National Hospital Organization Disaster Medical Center, Tokyo 190-0014, Japan; 22 Department of Emergency and Critical Care Medicine, St. Marianna University School of Medicine, Kanagawa 216-8511, Japan; 23 Department of Emergency and Critical Care Medicine, Nippon Medical School, Chiba Hokusou Hospital, Chiba 270-1694, Japan; 24 Department of Emergency and Critical Care Medicine, Nippon Medical School, Tama Nagayama Hospital, Tokyo 206-8512, Japan

**Keywords:** Acute lung injury, Hemodynamics, Pulmonary edema, Transpulmonary thermodilution, Vascular permeability

## Abstract

**Background:**

The features of early-phase acute respiratory distress syndrome (ARDS) are leakage of fluid into the extravascular space and impairment of its reabsorption, resulting in extravascular lung water (EVLW) accumulation. The current study aimed to identify how the initial EVLW values and their change were associated with mortality.

**Methods:**

This was a *post hoc* analysis of the PiCCO Pulmonary Edema Study, a multicenter prospective cohort study that included 23 institutions. Single-indicator transpulmonary thermodilution-derived EVLW index (EVLWi) and conventional prognostic factors were prospectively collected over 48 h after enrollment. Associations between 28-day mortality and each variable including initial (on day 0), mean, maximum, and Δ (subtracting day 2 from day 0) EVLWi were evaluated.

**Results:**

We evaluated 192 ARDS patients (median age, 69 years (quartile, 24 years); Sequential Organ Failure Assessment (SOFA) score on admission, 10 (5); all-cause 28-day mortality, 31%). Although no significant differences were found in initial, mean, or maximum EVLWi, Δ-EVLWi was significantly higher (i.e., more reduction in EVLWi) in survivors than in non-survivors (3.0 vs. −0.3 mL/kg, *p* = 0.006). Age, maximum, and Δ-SOFA scores and Δ-EVLW were the independent predictors for survival according to the Cox proportional hazard model. Patients with Δ-EVLWi > 2.8 had a significantly higher incidence of survival than those with Δ-EVLWi ≤ 2.8 (log-rank test, *χ*^2^ = 7.08, *p* = 0.008).

**Conclusions:**

Decrease in EVLWi during the first 48 h of ARDS may be associated with 28-day survival. Serial EVLWi measurements may be useful for understanding the pathophysiologic conditions in ARDS patients. A large multination confirmative trial is required.

## Background

Acute respiratory distress syndrome (ARDS) is characterized by life-threatening hypoxemia with high mortality rates [1]-[3]. In the early phase of ARDS (i.e., pathologically ‘exudative stage’), intravascular fluid leakage into the interstitium and alveoli of the lung due to diffuse alveolar damage results in accumulation of extravascular lung water (EVLW) [4],[5]. Recently, the transpulmonary thermodilution technique has facilitated bedside quantitative evaluation of EVLW, as well as its indexed value extravascular lung water index (EVLWi, mL/kg), with robust validation in both accuracy and precision [6]-[9]. Consistent with the pathological concept, recent studies indicated that the EVLWi value represented the severity of lung injury in ARDS patients [10]-[13]. Moreover, previous studies suggested that increasing absolute EVLWi values in the early phase of ARDS (i.e., increase in initial EVLWi [14],[15], mean EVLWi for the first 3 days [16], and EVLWi on day 2 [12]) were associated with patient mortality.

Reabsorption of EVLW can also be impaired during the early phase of ARDS in addition to the primary leakage of fluid to the extravascular space [4],[17]. The landmark study by Ware and Matthay [18] showed that impaired alveolar fluid clearance early in the course of ARDS was well correlated with poor clinical outcome. Consistent with these pathophysiological disturbances (i.e., impaired reabsorption of EVLW), changes in EVLWi (Δ-EVLWi) in the early phase of acute respiratory failure may constitute a significant predictor of survival [19],[20].

Cordemans et al. [20] previously reported that the maximum difference between EVLWi measurements during the patient's stay in the intensive care unit (ICU) was related to poor prognosis. More recently, Jozwiak et al. [21] reported that the maximum value of EVLWi over the entire ARDS episode was an independent risk factor for mortality. Thus, continuous leakage due to persistent precipitating underlying causes and/or impairment of EVLW reabsorption may result in a poor outcome [20]-[22]. However, the maximum EVLWi value reached during an ICU stay or ARDS episode cannot be predicted on a given day in clinical practice; thus, evaluating changes in early EVLWi might be more useful for accurate evaluation of the pathophysiological condition [21].

Therefore, the aim of the current study was to identify how the initial EVLW values and their changes were associated with mortality in adult ARDS patients by re-analyzing a large multicenter cohort study database.

## Methods

### Design and patients

The current study was a *post hoc* analysis of the PiCCO Pulmonary Edema Study, a multicenter prospective cohort study that examined respiratory-distressed patients admitted to 23 participating institutions in Japan [10],[23]-[26]. This study was approved by the ethics committees of all 23 institutions, and written informed consent was obtained from all patients or their next of kin.

The primary inclusion criteria were age of >15 years (no upper age limit), mechanical ventilation (expected duration, >48 h) required for acute respiratory failure with a PaO_2_/FiO_2_ ratio of ≤300 mmHg, and bilateral infiltration on chest radiography. An EVLWi of >10 mL/kg was used to define pulmonary edema, in accordance with definitions in previous reports [8],[10],[13],[27]. Exclusion criteria were as follows: >5 days from the onset of acute respiratory failure; chronic respiratory insufficiency; history of pulmonary resection, pulmonary thromboembolism, or severe peripheral arterial disease; cardiac index of <1.5 L/min/m^2^; lung contusion; or burns as well as other causes rendering the patient unsuitable for evaluation with the transpulmonary thermodilution technique [23].

The pathophysiological differential diagnosis for respiratory insufficiency was performed by at least three experts (specializing in intensive care, respirology, and cardiology), who retrospectively determined the pathophysiological mechanism of respiratory insufficiency as (a) cardiogenic (hydrostatic) pulmonary edema, (b) permeability pulmonary edema (i.e., ARDS), or (c) pleural effusion with atelectasis but no evidence of lung edema secondary to increased hydrostatic pressure or vascular permeability as previously described [23]. For this purpose, the experts carefully scrutinized the patient's medical history, clinical presentation and course, and findings of chest computed tomography, radiography, and echocardiography. They also considered the time course of all the preceding findings, including daily fluid intake and output, and the balance thereof, and requirement of systemic management and respiratory therapy. The hospital type was categorized as academic or non-academic. Hospital volume was defined as the number of patients that participated in the current analysis and was categorized into tertiles (i.e., low, medium, and high).

We considered the increased permeability pulmonary edema group (i.e., (b) above) as ARDS [10],[23] and included the corresponding patients in the current study. At the time of enrollment (day 0), the patient was evaluated with regard to clinical condition, cause of respiratory insufficiency, Sequential Organ Failure Assessment (SOFA) score [28], and echocardiography chest computed tomography. Blood samples were obtained via the arterial catheter at the same time as thermodilution measurements were performed.

### Thermodilution measurements

A 4- or 5-French arterial thermistor-tipped catheter (PV2014L16N, PV2014L22N, or PV2015L20N; Pulsion Medical Systems, Munich, Germany) was inserted in all patients and connected to a PiCCO® monitoring system (PiCCO Plus system or PiCCO 2 system) or Philips IntelliVue monitor (Philips Medical Systems, BG Eindhoven, The Netherlands) equipped with a PiCCO technology module. Previous reports have discussed the principles and validation of these single-indicator transpulmonary thermodilution-derived variables [20],[29],[30]. In short, a 15-mL bolus of cold normal saline was injected through a central venous catheter. The thermodilution curves were then recorded from the thermistor of the PiCCO catheter to allow for estimation of cardiac output, global end-diastolic volume (GEDV) [31], EVLW, pulmonary vascular permeability index (PVPI) [23], global ejection fraction, and systemic vascular resistance index. The principles and validation of these single-indicator transpulmonary thermodilution-derived variables have been discussed in detail previously [32],[33]. We collected the data of absolute EVLW value. The absolute EVLW value was indexed to predicted body weight, calculated as 50 + 0.91 (height (cm) − 152.4) for males and 45.5 + 0.91 (height (cm) − 152.5) for females [23],[34]. For indexing EVLW, the predicted body weight (EVLWi; normal range, 7.4 ± 3.3 mL/kg) instead of the actual body weight was used because the EVLWi has been shown to be a better prognostic indicator than EVLW indexed to the actual body weight [8],[11],[14],[16],[35]. Measurements were performed every 24 h for 3 days. The intervention and treatment were decided by the attendant doctors at each institution, most of which follow the Japanese ARDS guidelines ‘Guideline for ALI/ARDS diagnosis and treatment’ (second edition) [36]. The PiCCO® system was only used for passive monitoring, and no treatment algorithm based on parameters obtained with transcardiopulmonary thermodilution was used.

### Outcomes

The principal outcome measures were survival within 28 days and the initial, mean, maximum, and Δ-EVLWi (subtracting EVLWi day 2 from day 0).

### Statistical analysis

Data were expressed as mean (±SD) or median (quartile), as appropriate. Patients were divided into two groups according to survival at day 28. Continuous variables were compared between the groups using the *t* test or Mann-Whitney *U* test for continuous variables, as appropriate. Categorical variables were analyzed using the chi-square test or Fisher's exact test. Areas under the receiver operator characteristic (ROC) curves (AUC) for SOFA and EVLWi variables (i.e., initial, mean, maximum, and Δ) to predict 28-day survival were calculated. The Youden index was used to determine the cutoff value for the variables regarding 28-day survival. Cox proportional hazard analysis (forward stepwise methods) was used to estimate hazard ratio after accounting for *a priori* factors (age, sex, and cumulative fluid balance) and other variables of *p* < 0.15 based on univariate analyses as the selection criterion. Patients were then divided into two groups according to the cutoff value estimated by the Youden index, and survival analysis was performed using Kaplan-Meier plots with log-rank statistics between the groups. All data were analyzed using SPSS 22.0 for Windows (IBM, Armonk, NY, USA) and Stat Flex 6.0 for Windows (Artech, Osaka, Japan); *p* < 0.05 was considered significant.

## Results

### Patients

**Figure 1:**
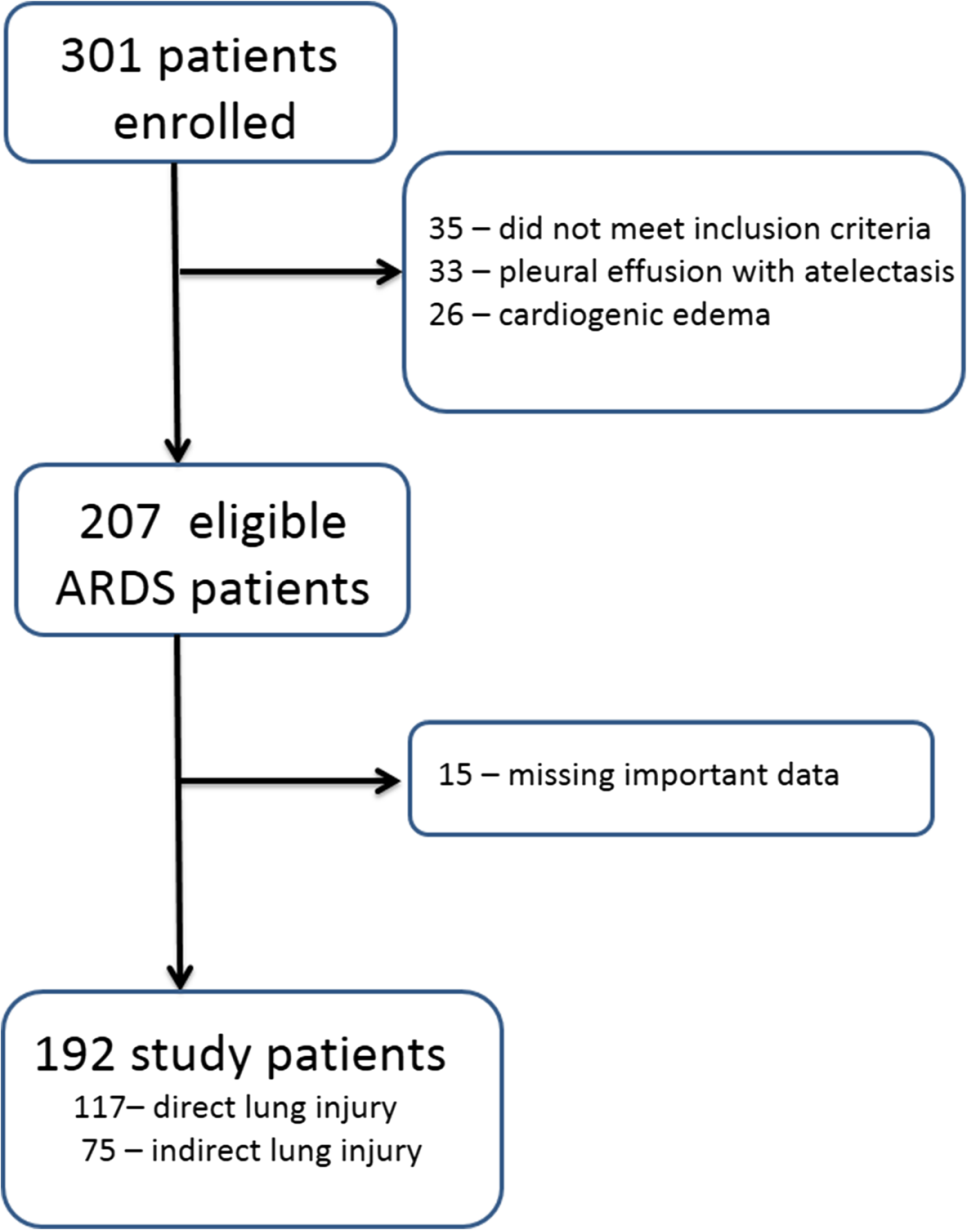
**Flow diagram of patient enrollment.** Missing important data: extravascular lung water index data missing either on day 0, day 1, or day 2.

From March 2009 to August 2011, 301 patients with respiratory insufficiency were enrolled in the PiCCO Pulmonary Edema Study [23]. Of the 301 patients initially recruited, 192 matched the inclusion criteria for this analysis (Figure 1). Overall, the median age of the patients was 69 (24) and the initial SOFA score was 10 (5). Fifty-nine (30.7%) patients died within 28 days after admission. The 192 patients were separated into survivors and non-survivors at day 28 (Table 1). The survivors had significantly lower SOFA scores, lower creatinine levels, and lower cumulative fluid balance values (Table 1).

**Table 1:** Patient characteristics according to survival at day 28

**Patient characteristics**	**Survivors (*n*** **= 133)**	**Non-survivors (*n*** **= 59)**	***p*** **value**
Sex (male), *n* (%)	88 (66.2)	37 (62.7)	0.64
Age, years	68 (23)	72 (19)	0.05
Hospital type (academic), *n* (%)	87 (65.4)	33 (55.9)	0.26
Hospital volume, *n* (%)			0.95
≤8	44 (33.1)	20 (33.9)	
9 to 14	46 (34.6)	19 (32.2)	
≥14	43 (32.3)	20 (33.9)	
Direct lung injury, *n* (%)	78 (58.6)	39 (66.1)	0.39
SOFA score on day 0	10 (5)	11 (5)	<0.001
Mean SOFA score for 3 days, mean ± SD	9.6 ± 4.5	11.3 ± 4.3	<0.001
Maximum SOFA score	11 (5)	13 (5)	<0.001
Δ-SOFA score, mean ± SD	0.89 ± 2.1	0.53 ± 2.3	<0.001
Mean arterial pressure, mmHg	76 (24)	77 (22)	0.61
Central venous pressure, mmHg	10 (6)	10 (9)	0.89
PaO2/FiO_2_, mmHg	151.4 (112)	138.1 (103)	0.14
Serum albumin, mg/dL	2.5 (0.9)	2.6 (0.8)	0.84
Serum creatinine, mg/dL	0.9 (1.1)	1.4 (1.4)	0.003
Diuretic use, *n* (%)	68 (51.1)	28 (47.4)	0.64
Renal replacement therapy, *n* (%)	32 (24.1)	21 (35.6)	0.10
Steroid use, *n* (%)	49 (36.8)	24 (40.7)	0.61
Use of vasopressors, *n* (%)	91 (68.4)	44 (74.6)	0.39
Cumulative fluid balance over 48 h, mL	3,015 (4,918)	4,595 (6,187)	0.02

Data were shown in median (quartile) unless otherwise presented. Hospital volume was defined as the number of patients that participated in the current analysis and was categorized into tertiles (i.e., low, medium, and high). SOFA, Sequential Organ Failure Assessment.

### Thermodilution measurement variables

**Figure 2:**
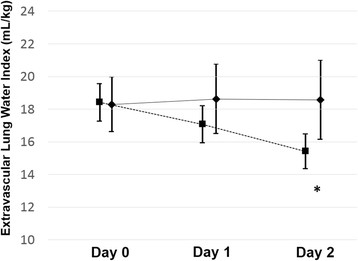
**Comparison of serial EVLWi in survivors (square) and non-survivors (diamond).** Error bar indicates 95% confidence interval. *p = 0.006.

Although there was no significant difference between the survivors and non-survivors with respect to initial, mean, maximum, or Δ-GEDV nor initial, mean, or maximum-EVLWi, significant differences existed for Δ-EVLWi (Table 2). Comparison of serial EVLWi in survivors and non-survivors is presented in Figure 2. Furthermore, 71% of the survivors exhibited an improvement (increase) in Δ-EVLWi, compared with 54% of the non-survivors (*p* = 0.02).

**Table 2:** Transpulmonary thermodilution data during the study period according to survival at day 28

**Patient characteristics**	**Survivors (*n*** **= 133)**	**Non-survivors (*n*** **= 59)**	***p*** **value**
GEDVi on day 0, mL/m^2^	825 ± 212	808 ± 199	0.58
Mean GEDVi for 3 days, mL/m^2^	835 ± 197	832 ± 191	0.92
Maximum GEDVi, mL/m^2^	937 ± 251	932 ± 216	0.90
Day of maximum GEDVi measurement, *n* (%)			0.45
Day 0	40 (30.1)	18 (30.5)	
Day 1	45 (33.8)	15 (25.4)	
Day 2	48 (36.1)	26 (44.1)	
Δ-GEDVi, mL/m^2^	−29 ± 198	−63 ± 199	0.26
EVLWi on Day 0, mL/kg	18.4 ± 6.7	18.3 ± 6.5	0.90
Mean EVLW for 3 days, mL/kg	17.0 ± 5.4	18.5 ± 7.2	0.11
Maximum EVLW, mL/kg	20.7 ± 7.3	21.8 ± 9.2	0.36
Day of maximum EVLWi measurement, n (%)			0.14
Day 0	64 (48.1)	21 (35.6)	
Day 1	49 (36.8)	23 (39.0)	
Day 2	20 (15.0)	15 (25.4)	
Δ-EVLWi, mL/kg	3.0 ± 7.4	−0.3 ± 7.6	0.006
PVPI on Day 0	3.2 ± 1.3	3.2 ± 1.4	0.98
Mean PVPI for 3 days	2.9 ± 1.0	3.1 ± 1.3	0.21
Maximum PVPI	3.6 ± 1.4	3.8 ± 1.8	0.40
Day of maximum PVPI measurement, *n* (%)			0.17
Day 0	76 (57.1)	25 (42.4)	
Day 1	36 (27.1)	22 (37.3)	
Day 2	21 (15.8)	12 (20.3)	
Δ-PVPI	0.6 ± 1.3	0.2 ± 1.4	0.06

EVLWi, extravascular lung water index; GEDVi, global end-diastolic volume index; PVPI, pulmonary vascular permeability index; Δ-EVLWi, reduction in EVLWi from day 2 to day 0; Δ-GEDVi, reduction in GEDVi from day 2 to day 0; Δ-PVPI, reduction in PVPI from day 2 to day 0.

### ROC analysis

AUC of ROC analyses to predict 28-day survival for each SOFA variable, i.e., initial, mean, maximum, and Δ-SOFA, were 0.65 (95% CI, 0.57 to 0.73, *p* = 0.001), 0.71 (0.64 to 0.79, *p* < 0.001), 0.70 (0.63 to 0.78, *p* < 0.001), and 0.66 (0.58 to 0.74, *p* < 0.001), respectively. There was no significant AUC difference among the SOFA variables.

AUC of ROC analyses to predict 28-day survival for each EVLWi variable, i.e., initial, mean, maximum, and Δ-EVLWi, were 0.50 (0.41 to 0.59, *p* = 0.98), 0.55 (0.46 to 0.64, *p* = 0.29), 0.52 (0.42 to 0.61, *p* = 0.72), and 0.62 (0.53 to 0.70, *p* = 0.01), respectively. Although the value of AUC for Δ-EVLWi was higher than that for initial-EVLW, it did not reach a statistically significant level (0.62 vs. 0.50, *p* = 0.06).

**Figure 3:**
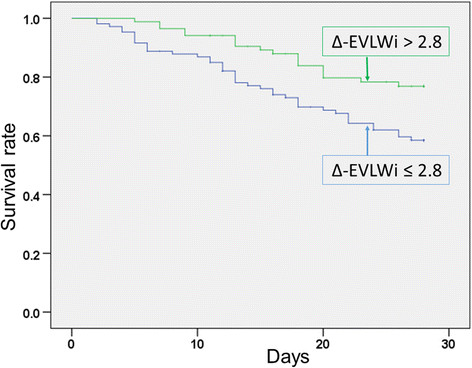
**Kaplan-Meier survival curves for patients that were categorized by** Δ**-EVLWi > 2.8 or** Δ**-EVLWi ≤ 2.8.** Significant differences were found between the two groups (log-rank test: *χ*^2^ = 7.08, *p* = 0.008).

The Δ-EVLWi threshold that was best associated with 28-day mortality was 2.8 as estimated by the Youden index (70% sensitivity, 51% specificity, 39% positive predictive value, 79% negative predictive value). Patients with Δ-EVLWi > 2.8 had a significantly higher incidence of survival than the patients with Δ-EVLWi ≤ 2.8 (chi-square test, 78.8% vs. 61.7%, *p* = 0.01). The Kaplan-Meier survival curve showed a significant time-dependent difference between patients with Δ-EVLWi > 2.8 and Δ-EVLWi ≤ 2.8 during 28 days (log-rank test, *χ*^2^ = 7.08, *p* = 0.008) (Figure 3).

### Cox proportional hazard model analysis

Factors as a priori and in accordance with the results of the univariate analyses as presented in Tables 1 and 2, age; sex; cumulative fluid balance; initial-, mean-, maximum-, and Δ-SOFA scores; serum creatinine level; use of renal replacement therapy; mean- and Δ-EVLWi; and Δ-PVPI were entered (*p* < 0.15 on univariate analysis) in the Cox proportional hazard model for 28-day survival. Age; maximum- and Δ-SOFA scores; and Δ-EVLWi were independently associated with survival (Table 3).

**Table 3:** Cox proportional hazards model analysis for the prediction of 28-day survival

	**Hazard ratio (95%CI)**	***p*** **value**
Age	1.02 (1.00 to 1.04)	0.02
Sex	0.99 (0.57 to 1.7)	0.97
Cumulative fluid balance	1.0 (0.99 to 1.0)	0.28
Δ-EVLWi	0.95 (0.92 to 0.98)	0.006
Maximum SOFA score	1.2 (1.1 to 1.4)	<0.001
Δ-SOFA score	0.80 (0.71 to 0.91)	<0.001

Δ-EVLWi, reduction in extravascular lung water index from day 2 to day 0; SOFA, Sequential Organ Failure Assessment; Δ-SOFA, reduction in SOFA score from day 2 to day 0.

## Discussion

The current study suggested that change in EVLWi during the first 48 h - and not initial, mean, or maximum EVLWi value - was associated with 28-day survival. In addition, Δ-EVLWi was one of the independent factors that was associated with the time-dependent influence on survival even after adjustment for age and maximum and Δ-SOFA scores. Δ-EVLWi may represent the pathophysiologic status of ARDS recovery.

The strength of the current study was that we re-evaluated prospectively collected data from 23 institutions with relatively large sample data. Although the EVLW appears a promising variable for prediction of prognosis in ARDS patients and representation of the pathophysiologic state of ARDS, relevant previous studies were all single-center (or two-center) studies [12],[14]-[16],[19]-[21], and most included only a limited number of patients [12],[14]-[16],[19]. On the other hand, the current results suggest that Δ-EVLWi alone might not be adequate as a practical and useful parameter to predict mortality. Although higher than the AUC for mortality of the American-European Consensus Conference (AECC) definition or recently revised Berlin definition (0.536; 95% CI, 0.520 to 0.553 and 0.577; 95% CI, 0.561 to 0.593, respectively) [1],[37], the predictive value of AUC for mortality for Δ-EVLWi was only 0.62 in the present study. In addition, corresponding sensitivity, specificity, and positive/negative predictive values for Δ-EVLWi threshold that was best associated with 28-day mortality were relatively low. Moreover, effect size of Δ-EVLWi was small with a hazard ratio of 0.95 according to the Cox analysis. However, the main interest of the current study was the validation of the transpulmonary thermodilution-derived EVLWi, which not only predicts mortality but also may represent pathophysiological condition in the early phase of ARDS.

Mortality rates in ARDS are greatly influenced by the age of the patient and presence of non-pulmonary organ dysfunctions, and patients die more commonly from multiple organ failure than respiratory failure [2],[38]. Therefore, a scoring system that represents organ dysfunctions - the SOFA score - and its changes over time must be good predictors of survival in ARDS. The results of the current study revealed that maximum and Δ-SOFA scores were associated with the prognosis in clinical situations, which was consistent with the previous study [28]. Importantly, the current results showed that Δ-EVLWi was also an independent predictor of survival even after adjustment for age and maximum and Δ-SOFA scores. Thus, our results suggest that ARDS patients with potential multiple organ failure had comorbid conditions that hindered EVLW improvement. This phenomenon is consistent with the results of previous studies that revealed that compromised reabsorption of fluid in extravascular space is common in ARDS patients and is correlated with poor clinical outcome [4],[17],[18]. Conversely, Δ-EVLWi may represent the pathophysiologic status of recovery in ARDS [12],[20]-[22]. Our results were similar to the findings obtained by Cordemans et al. [20], who reported that the maximum difference between EVLWi measurements during the ICU stay was related to poor prognosis. Because maximum EVLWi cannot be identified in a given day, we defined Δ-EVLWi as the difference between the values at day 2 and day 0 (48 h) in the current study. We therefore believe that serial measurements of the EVLWi value and simple calculation of Δ-EVLWi in the first 48 h may aid understanding of the pathophysiological condition in ARDS.

Our data are inconsistent with previous ARDS studies that reported the prognostic value of initial, mean, or maximum EVLWi value [14],[16],[19],[21]. A possible explanation for the selection of initial and mean EVLWi as significant variables associated with prognosis may be an effect of the inclusion criteria used in those previous studies [12],[14],[16],[21], where ARDS was diagnosed on the basis of the AECC criteria [37]. Although the AECC criteria are simple and widely used, significant criticisms have been reported in terms of their diagnostic validity [1],[13],[27],[39],[40]. Martin et al. [41] suggested that 1/3 of the patients fulfilling the AECC criteria did not have elevated EVLWi, and these patients had improved survival compared with patients with increased EVLWi. As a result, patients with a wide variety of EVLWi values were entered in the previous ARDS studies, which might have contributed to detection of the initial or mean EVLWi value as a good prognostic factor even in a small sample size [12],[14],[16]; approximately 1/4 of the studied patients were reported as having an EVLWi of <7 mL/kg [12] or <10 mL/kg [14], which were considered within normal range [8],[13],[27],[42]. Therefore, these patients may not have had ARDS pathologically because a recent study suggested that an EVLWi of >10 mL/kg constituted the quantitative discriminating threshold for the diagnosis of diffuse alveolar damage [13]. Thus, patients with high initial EVLWi (with diffuse alveolar damage) were assumed to have poor outcome, which was consistent with the findings reported among critically ill patients in general (i.e., with heterogenous groups of patients) [33],[43].

A recent large retrospective single-center study of 200 ARDS patients by Jozwiak et al. [21], only 2% of whom had an EVLWi of <7 mL/kg, also suggested that there was no difference in initial EVLWi value between non-survivors and survivors (17 ± 9 mL/kg vs. 16 ± 7 mL/kg, *p* = 0.25) [21]. In the current study, only ARDS patients whose EVLWi was >10 mL/kg were included, which resulted in a similar initial EVLWi value in non-survivors and survivors (18.3 ± 6.5 mL/kg vs. 18.4 ± 6.7 mL/kg, *p* = 0.90). Interestingly, although there was no difference in the initial EVLWi between non-survivors and survivors, significant reductions in EVLWi were documented in survivors after 48 h in both studies, suggesting improvement of Δ-EVLWi. In the current study, Δ-EVLWi was the only significant and meaningful variable associated with 28-day prognosis on ROC analysis, which was confirmed by the subsequent Cox regression analysis and log-rank test. Thus, more attention must be paid to Δ-EVLWi among initially high EVLWi patients in present clinical practice and in future studies.

Maximum EVLWi value was not a significant variable associated with prognosis in the current study; this result differs from that of the study by Jozwiak et al. [21], despite both studies having similar sample size and initial EVLWi value. The main reason for this discrepancy may be the duration of the thermodilution evaluation period. Although Jozwiak et al. [21] studied the whole ARDS period (median duration of 12 days interquartile range, [7]-[21]) to determine maximum EVLWi, we evaluated data only for the first 3 days (48 h), which were considered to represent the early phase clinically and the ‘exudative stage’ pathologically. Thus, maximum EVLWi might have appeared after the early phase of ARDS in the current study. However, a physician performing a bedside EVLWi measurement in an ARDS patient cannot predict when the EVLW value will reach the maximum. On the other hand, evaluation of Δ-EVLWi after the first 48 h of intensive care is easily performed and a practical method to incorporate into daily clinical practice.

There are several limitations regarding the current study. First, 15 patients were excluded from the analysis because EVLWi data were missing either on day 0, day 1, or day 2. Second, this was a *post hoc* analysis of a prospective multicenter study from 23 institutions. Intervention and treatment were decided by the attendant doctors at each institution, and no treatment algorithm using parameters obtained with transcardiopulmonary thermodilution was used. We could not evaluate center effect on outcome because of the small sample size for each of the institutions. In addition, we do not know whether aggressive diagnostic and therapeutic interventions may change the outcome after recognition of worsening Δ-EVLWi in the early phase of ARDS. The presence of hemodynamic instability and need for fluid resuscitation may have a significant impact on Δ-EVLWi. We analyzed only 3-day EVLWi data; thus, effective fluid de-resuscitation might not yet have started. Further interventional studies, including EVLWi-driven management protocols, are required. Third, PVPI and changes in PVPI were not different between survivors and non-survivors incompatible with the results of previous studies [10],[21],[44]. This inconsistency may be explained by the small sample size of the selected patients and the imbalance between the survivors and non-survivors (the number of survivors was more than double the number of non-survivors). Although it did not reach a statistically significant level, Δ-PVPI was higher in survivors than non-survivors (0.6 vs. 0.2, *p* = 0.06). Because this was a retrospective *post hoc* analysis, we did not perform sample size estimation in advance for the current analysis. Fourth, even though mortality in critically ill patients with ARDS is influenced by various factors, we could not document the cause of death, day of ICU stay on which the patient was enrolled in the study, nor length of ICU stay because these data were not available in the PiCCO Pulmonary Edema Study database. Thus, all these limitations should be addressed in the future by well-designed large-sample multination prospective study.

## Conclusions

Although the effect size was small in this study, Δ-EVLWi during the first 48 h of ARDS in patients with high initial EVLWi is easily calculated and may be related to 28-day mortality. Serial extravascular lung water measurements in the early phase of ARDS may be useful for the understanding of the pathophysiologic condition in ARDS patients. A large multination confirmative trial is required.

## Abbreviations

ARDS: acute respiratory distress syndrome

EVLW: extravascular lung water

EVLWi: extravascular lung water index

GEDV: global end-diastolic volume

GEDVi: global end-diastolic volume index

PVPI: pulmonary vascular permeability index

SOFA: Sequential Organ Failure Assessment

## Competing interests

Takashi Tagami, Nobuyuki Saito, and Shigeki Kushimoto received speaker honoraria from Tokibo Co., Ltd. (import trader of the PiCCO system) for educational lectures at Japanese scientific meetings. The remaining authors declare no conflicts of interest. This study was not funded or sponsored by any organization.

## Authors' contributions

All authors conceived and designed the study, wrote the study protocol, and acquired the clinical data for the PiCCO Pulmonary Edema Study. TT was responsible for the statistical analyses for the current study and the first draft of the manuscript. All authors amended and commented on the manuscript. All authors read and approved the final manuscript.
